# Telomeric Expression Sites Are Highly Conserved in *Trypanosoma brucei*

**DOI:** 10.1371/journal.pone.0003527

**Published:** 2008-10-27

**Authors:** Christiane Hertz-Fowler, Luisa M. Figueiredo, Michael A. Quail, Marion Becker, Andrew Jackson, Nathalie Bason, Karen Brooks, Carol Churcher, Samah Fahkro, Ian Goodhead, Paul Heath, Magdalena Kartvelishvili, Karen Mungall, David Harris, Heidi Hauser, Mandy Sanders, David Saunders, Kathy Seeger, Sarah Sharp, Jesse E. Taylor, Danielle Walker, Brian White, Rosanna Young, George A. M. Cross, Gloria Rudenko, J. David Barry, Edward J. Louis, Matthew Berriman

**Affiliations:** 1 Wellcome Trust Sanger Institute, Wellcome Trust Genome Campus, Hinxton, United Kingdom; 2 The Rockefeller University, New York, New York, United States of America; 3 Institute of Genetics, The University of Nottingham Queen's Medical Centre, Nottingham, United Kingdom; 4 Department of Statistics, University of Oxford, Oxford, United Kingdom; 5 The Peter Medawar Building for Pathogen Research, University of Oxford, Oxford, United Kingdom; 6 Wellcome Centre for Molecular Parasitology, University of Glasgow, Glasgow, United Kingdom; University of Liverpool, United Kingdom

## Abstract

Subtelomeric regions are often under-represented in genome sequences of eukaryotes. One of the best known examples of the use of telomere proximity for adaptive purposes are the bloodstream expression sites (BESs) of the African trypanosome *Trypanosoma brucei*. To enhance our understanding of BES structure and function in host adaptation and immune evasion, the BES repertoire from the Lister 427 strain of *T. brucei* were independently tagged and sequenced. BESs are polymorphic in size and structure but reveal a surprisingly conserved architecture in the context of extensive recombination. Very small BESs do exist and many functioning BESs do not contain the full complement of expression site associated genes (*ESAG*s). The consequences of duplicated or missing *ESAG*s, including *ESAG9*, a newly named *ESAG12*, and additional variant surface glycoprotein genes (*VSG*s) were evaluated by functional assays after BESs were tagged with a drug-resistance gene. Phylogenetic analysis of constituent *ESAG* families suggests that BESs are sequence mosaics and that extensive recombination has shaped the evolution of the BES repertoire. This work opens important perspectives in understanding the molecular mechanisms of antigenic variation, a widely used strategy for immune evasion in pathogens, and telomere biology.

## Introduction

Subtelomeres are dynamic and fast-evolving regions of eukaryotic genomes owing to their remarkable plasticity [1]–[3]. Recombination between internal repeats and chromosomal arms results in the accumulation of species-specific sequences, commonly mediating adaptation to the environment. Despite their extreme genetic diversity, common aspects of structure and function are shared across telomeres from a diverse range of organisms [4].

The ability to encode contingency functions within their subtelomeric regions has been exploited by the African Trypanosome, subspecies of which cause the debilitating disease Human African Trypanosomiasis and the related disease ‘nagana’ in livestock [reviewed in [2]. *Trypanosoma brucei* evades the mammalian host's immune response by periodically changing its variant surface glycoprotein (VSG) coat (reviewed in: [5]), a dense monolayer of 5×10^6^ identical VSG dimers. The expressed *VSG* is located in specialised subtelomeric transcription units known as the Bloodstream Expression Sites (BES). The total number of BESs is dependent on the subspecies and strain but is believed to be about 20 for the *T. brucei* Lister 427 strain [6] used in the present study. To change the VSG coat, transcription is switched to an alternative *VSG* by recombination of a new *VSG* into the active BES or by transcriptional silencing of one BES with concomitant activation of a second.

Sequencing of a few BESs has revealed a diverse range of 11 polymorphic genes, called Expression Site Associated Genes (*ESAG*s) [7], few of which have been functionally characterised: *ESAG3*, *ESAG5* and *ESAG11* encode membrane-associated or membrane-targeted proteins [8]; *ESAG4* encodes a putative transmembrane receptor with adenylate cyclase activity [9]; *ESAG6* and *ESAG7* encode subunits of a heterodimeric receptor that mediates uptake of host transferrin [10], [11]; *ESAG8* encodes a putative nuclear DNA-binding protein [12], [13]; *ESAG10* encodes a homologue of the *Leishmania* biopterin transporter [14] whilst *ESAG9* [15] has an unknown function. As many *ESAG*s are members of large families and are dispersed in the genome [16]–[18], and as a low level of transcription occurs even from ‘inactive’ BESs, determining the precise function of individual *ESAG*s in active expression sites is challenging.

In an effort to systematically analyse the BES repertoire of a single clone, a library of transformation-associated recombination (TAR) clones was made [19]. In the present study, sequence analysis revealed that the 19 TAR clones fall into 14 distinct BES groups. Independently, 13 randomly tagged BESs were constructed and analyzed, confirming the structures and re-defining the minimal gene complement of a functional BES for survival in culture.

Patterns of sequence variation between different *ESAG* families were analysed revealing that expression sites are sequence mosaics - related to each other differently depending on the gene family examined - and highlighting the significant role that recombination between gene families has played in forming BES sequences. In characterising a complete BES repertoire these results provide a perspective on nucleotide diversity, but also on the mechanisms regulating their origins and diversity.

## Results

Two independent approaches were undertaken. First, BESs were randomly tagged with a G418-resistance gene, and we will refer to these clones as ‘*NEO*-tagged’. Second, TAR-cloned BESs were sequenced, and we will refer to these clones either by their TAR clone number or by the BES to which they have been assigned.

### Tagging BESs

*T. brucei* cell lines tagged with two drug-resistance markers were generated to select parasites that had stochastically activated an individually tagged BES from the background of highly similar BESs. The active BES1 was tagged with a Puromycin resistance gene (*PUR*), immediately downstream of the promoter. In the remaining silent BESs, transcription initiates at the BES promoter but is rapidly attenuated. This low level of transcriptional activity allowed the integration of a Neomycin resistance gene (*NEO*) immediately downstream of a second ‘silent’ BES promoter. In this way, we obtained 30 double-tagged clones. Each randomly *NEO*-tagged BES was typed according to its *ESAG6* hypervariable region, the distance between the *ESAG6* gene and the promoter, the number of BES promoters, and the sequence of the expressed *VSG* upon BES activation. Analysis of the 30 *NEO*-tagged clones and comparison to the TAR clones showed that 13 different BESs were tagged: BES1, 2, 3, 4, 5, 7, 8, 12, 13, 14, 15a and 15b (a duplicated BES, see below) and 17 (Tables 1 and 2). All tagged BES could be activated *in vitro*, except for BES8. Characterisation of these clones confirmed many of the apparently anomalous structures revealed by the TAR sequences (see below). The only two cloned BESs that were not tagged were BES10 and BES11 (Tables 1 and 2).

**Table 1:** BES overview.

BES	TAR clone sequenced	*Nomenclature of VSG* genes			Deviation from conserved architecture			*Drug-resistance* tagged clone	*NEO*-tagged clone consistent with sequenced TAR clone	BES could be activated *in vitro*	Genome localisation: type and size (Mb) of chromosome
		*2008*	*Lab-specific*	*MITat*	Multiple copies of ESAGs?	Missing ESAGs?	Other				
1 (a)	40	427-2	221	MITat 1.2	large-scale	-	70 bp-repeat array (×2)	Yes	-	Yes	MBC-3.0
2 (a)	129	427-9	VO2	MITat 1.9	7 (×2)	-	ingi element 3′ of 70-bp repeat	Yes	Yes	Yes	IC-0.325
3	2, 15	427-6	121	MITat 1.6	-	-		Yes	Yes	Yes	MBC-2.05
4	3, 28	427-21	T3	MITat 1.21	7 (×3)	-	VSG ψ 3′ of 70-bp repeat	Yes	Yes	Yes	IC-0.250
5	98	427-18	800	MITat 1.18	-	-		Yes	No (c)	Yes	MBC-1.95
7	65 (b), 153	427-3	224	MITat 1.3	6 (×2)	7	*ESAG3* ψ 3′ of 70-bp repeat	Yes	Yes	Yes	MBC >3.1
8	64	427-14	-	MITat 1.14	-	1,2,3,4,5,6,8,9,11	Single *ESAG7* gene	Yes	No (c)	No	MBC >3.1
10	134	427-15	-	MITat 1.15	-	1,2,3,4,8,11	23 bp trace of 70-bp repeat	No	N/A	N/A	IC-0.450
11	122	427-16	-	MITat 1.16	4 (×2)	1,2,11	additional *VSG* ψ; copy of *ESAG9*	No	N/A	N/A	IC-0.180
12	29	427-8	OD1	MITat 1.8	-	3,4,8		Yes	Yes	Yes	MBC-1.7
13	56	427-17	JS1	MITat 1.17	8 (×2)	-		Yes	Yes	Yes	IC-0.180
14	10	427-19	-	MITat 1.19	-	3,4,8	2 additional *VSG* ψs; 70 bp-repeat array (×2)	No	N/A	N/A	N/A
	117 (not sequenced)	427-8	OD1	MITat 1.8	N/A	N/A	N/A	Yes	Yes	Yes	MBC >3.1
15 (two copies: 15a, 15b)	126	427-11	bR-2	MITat 1.11	-	-	*ESAG8(×2)*; *VSG* ψ (×2); *ESAG3* ψ (×2); 70 bp-repeat array (×2)	Yes (15a & 15b)	Yes	Yes	2 MBC: 1.35, 1.7
16	128 (d)	427-2	221	MITat 1.2	N/A	N/A	N/A	N/A	N/A	N/A	N/A
17 (a)	51 (b), 59	427-13	NA1	MITat 1.13	-	-	-	Yes	Yes	Yes	IC-0.295

In this paper, we propose a new simple and logical *VSG* nomenclature. The hyphenated name indicates the *T. brucei* strain, followed by a number that identifies each unique *VSG*. For very closely related (probably antigenically indistinguishable) members of a *VSG* family, the number may be followed by a lower-case letter (for example Lister 427-3). When no misunderstanding can occur between strains under discussion in a particular context, the strain information may be omitted and the *VSG* referred to only by its number (for example, VSG 3). ND: not determined; N/A: not applicable; IC: intermediate chromosome; MBC: Megabase chromosome. (a) BES1, 2 and 17 had been previously cloned in BACs and identified as 221 ES, VO2 ES and Bn-2 ES, respectively [27]. (b) TAR65 and TAR51 were re-classified as belonging to BES group 7 and 17, respectively, based on global alignments showing sequence identity along the length of the sequence. The only differences were found in the promoter region, the target site for recombination during construction of the library. (c) Restriction mapping of the NEO tagged BES suggests the existence of two promoters, whereas the TAR clone has a single promoter. (d) TAR clone 128 is likely to be a recombinant between expression sites belonging to BES groups 1 and 15 (see text).

**Table 2:** Summary of the number of TAR clones sequenced, BES identified and BES activated.

	Number	TAR clones or BES	TAR clones or BES missing
TAR clones	19	40, 129, 2, 15, 3, 28, 98, 65, 153, 64, 134, 122, 29, 56, 10, 126, 128, 51, 59	TAR128 is probably a recombination artifact
Unique BES	14	1, 2, 3, 4, 5, 7, 8, 10, 11, 12, 13, 14, 15, 17	TAR2 and TAR3 were duplicates of TAR15 and TAR28, respectively; TAR51 and TAR65 were re-classified
All BES	15	1, 2, 3, 4, 5, 7, 8, 10, 11, 12, 13, 14, 15a+15b, 17	BES15 is present in two copies in the genome
Tagged BES	13	1, 2, 3, 4, 5, 7, 8, 12, 13, 14, 15a+15b, 17	BES10 and BES11 were not tagged
Functional tagged BES	12	1, 2, 3, 4, 5, 7, 12, 13, 14, 15a+15b, 17	BES8 was tagged with NEO gene, but no G418-resistant clones could be obtained.

### Chromosomal localization of BESs

**Figure 1:**
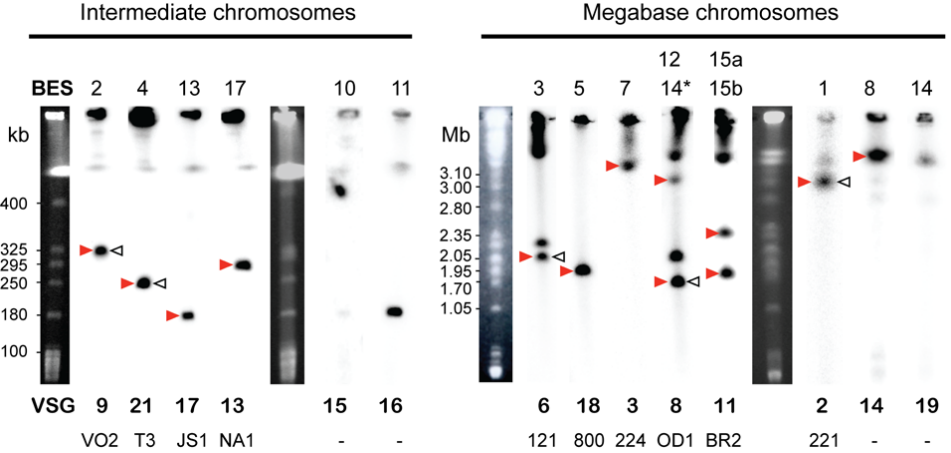
Chromosomal distribution of BES-resident *VSG*s. Red arrows indicate BES randomly tagged in this study with *NEO*. Open arrows indicate BES activated by [58] and subsequently tagged. Names of *VSG* genes are indicated at the bottom, using the newly proposed strain-specific numbering (top row) and lab-specific names (bottom row). BES10 and 11 were not tagged in either study. BES14 is represented in two lanes: 14* refers to TAR117 (which was not sequenced and harbours VSG8), whereas 14^$^ refers to TAR10 (which was sequenced and harbours VSG9).

The chromosome containing each BES (indicated by arrows in Figure 1) was identified by Southern blotting of PFGE-separated chromosomal DNA with *NEO* and *VSG* probes (Figure 1). It had been shown previously that BES2 is located on an intermediate chromosome [20]. We now show that BES4, BES10, BES11, BES13 and BES17 are also present in this chromosome class and that the megabase-sized chromosomes encode all remaining BES, consistent with previous reports [21].

In some cases, the *VSG* probe hybridized with more than one chromosome, indicating that the genome contains multiple copies of the corresponding *VSG*. For instance, BES12 and BES14 contain *VSG427-8*, and BES15a and BES15b contain *VSGbR-2/427-11*, on megabase chromosomes of different sizes (Figures S1A and S1B, respectively). *VSG121/427-6* is present as a non-BES copy (Dreesen O., personal communication).

### Switching mechanism and frequency

**Figure 2:**
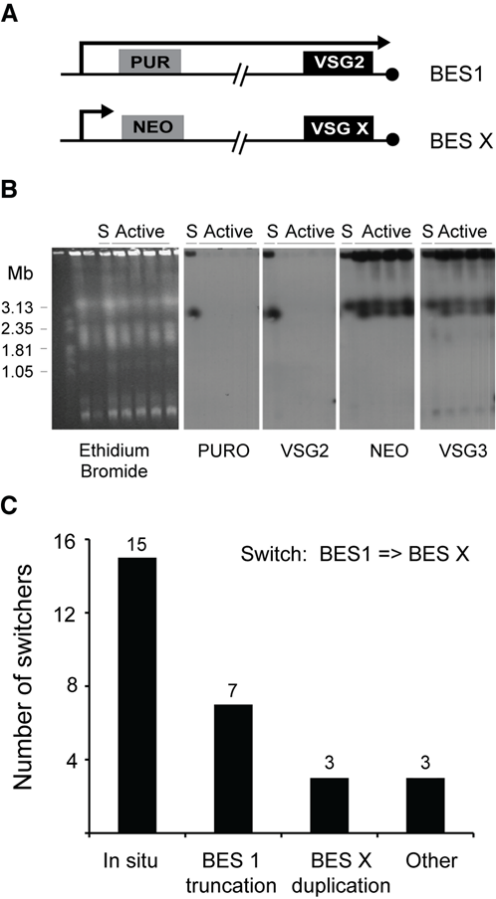
Types of switching mechanisms observed for tagged BES clones *in vitro*. A. *NEO*-tagged clones have PUR in BES1 and *NEO* in an unknown BES. B. Example of a BES duplication. PFGE analysis of a BES7-tagged clone (17.9), when this BES is silent (S) and after activation. *NEO* and *VSG3* genes were duplicated onto the chromosome that originally contained *PUR* and *VSG221/427-2*. C. Quantification of the frequency of the different types of mechanisms. “Other” refers to recombination events that involved more than 2 chromosomes.

The switching assay was designed to select for trypanosomes that had undergone an *in situ* switch by transcriptionally silencing BES1 and activating another BES without any DNA rearrangements. To assess the efficiency of selecting *in situ* switchers, Puromycin- and G418-resistant clones were analyzed by PFGE. If an *in situ* switch had occurred, the chromosomal locations of *PUR*, *NEO* and *VSG* should be unchanged: *PUR* and *VSG221/427-2* should be on chromosome VIa, whereas *NEO* and the newly identified *VSG* should be located on a different chromosome. PFGE analysis of 28 NEO-tagged clones that had activated different BES (2, 4, 5, 7, 13, 14, 15b and 17) confirmed that *in situ* switching was the predominant switching mechanism (15 switch events), but other switching mechanisms were also observed (Figure 2). In BES2- or BES15-tagged clones, for example, we observed that *VSG221/427-2* and *PUR* were deleted, whereas *NEO* and the new *VSG* remained in their original chromosomal location. This suggests that activation of BES5 and BES15 was accompanied by the truncation of BES1, a phenomenon previously reported for another BES [22]. In three of the 28 cases studied, the active BES was replaced by duplicative transposition of the entire new BES. In switchers that activated BES7 (Figure 2B), BES13 or BES15b, *VSG221/427-2* and *PUR* were lost and *NEO* and the new *VSG* genes were duplicated onto chromosome VIa. This switching mechanism has been previously detected in switchers obtained in mice [23] or by negative-selection *in vitro* [*24*]. In three cases, we observed unconventional recombination events that involved more than two chromosomes. These complicated VSG switch events that have undergone multiple DNA rearrangements have been previously documented [25].

In our cell-lines, the frequency of switching between BES1 and a second BES can be measured as the ratio between the number of G418-resistant clones and the total number of starting cells. Switching frequency was measured for clones in which the *NEO* gene was in BES4, 5, 13, 15b and 17. Consistent with previous reports, the average frequency was around 2±3×10^−6^ switchers/cell, with BES15 showing the lowest switching frequency (8.3×10^−8^ switchers/cell) [20], [26]. However, it should be noted that these measurements were based on a small number of independent clones (usually 3). A larger-scale study would be necessary to assess if the differences of switching frequency are statistically significant and how those correlate with the DNA sequence of the activated BES.

### Sequencing and assembly of TAR clones

**Figure 3:**
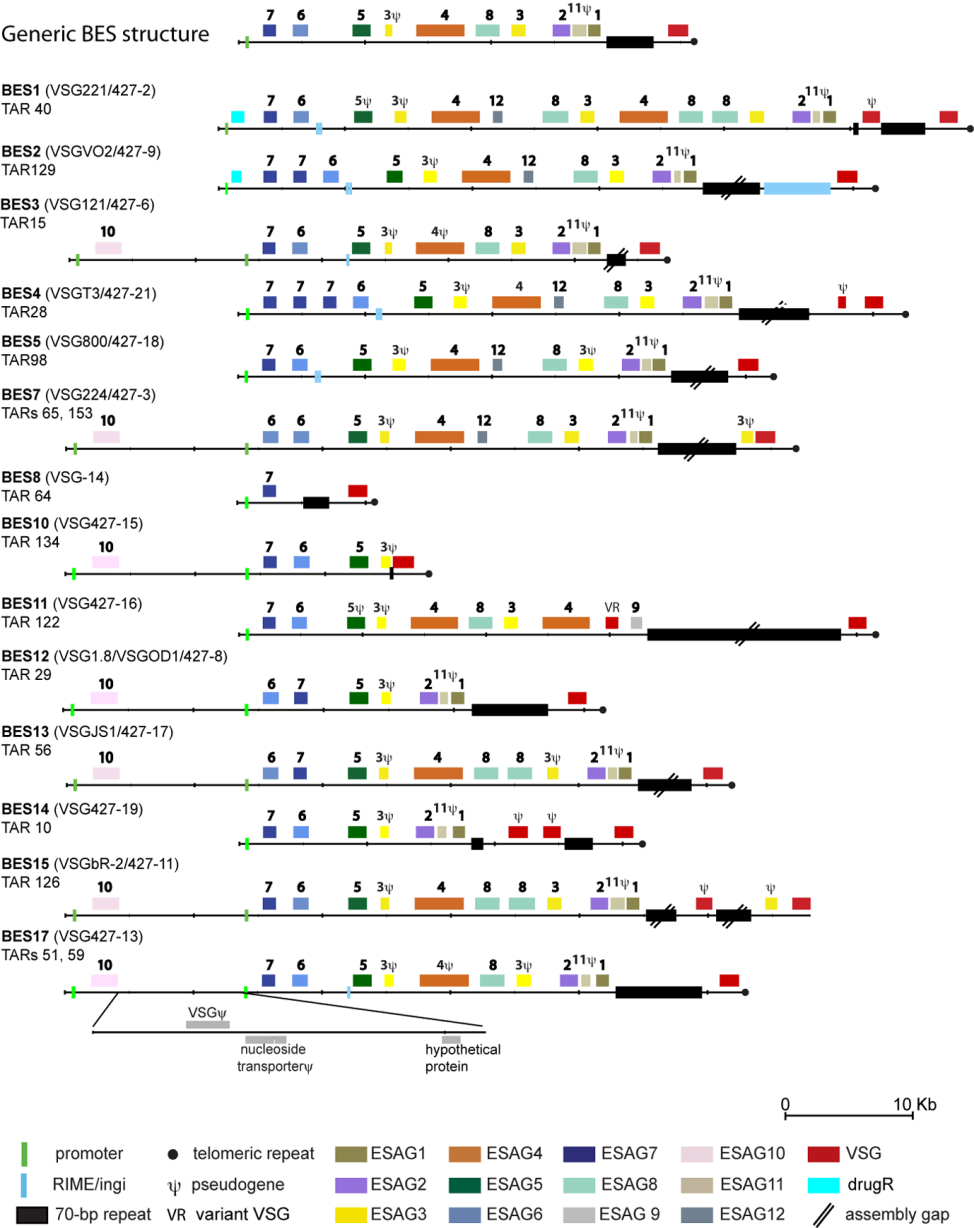
Overview of *T. brucei* Lister 427 BES. BES are drawn to scale and have been aligned at their 5′-most *ESAG7* or *ESAG6* sequence. The inset shows the regions conserved between the dual promoters present in some of the clones. The *VSG* indicated in front of the BES label refers to the telomere-proximal *VSG*.

Nineteen TAR clones [19] were sequenced, manually finished and analysed. This set represents 14 unique BESs, with TAR clones from BESs 9 and 16 having been re-classified (Table 2). For each BES group, at least one clone was sequenced (Figure 3, Table 1), choosing a larger sized clone wherever possible. Additional clones were sequenced for BES3 and BES4 to verify the sequence and clone integrity. Global alignments showed that TAR clones 2 and 15 (BES3) as well as 3 and 28 (BES4) are 100% identical at the DNA level with the exception of an *ESAG7* triplication in TAR28. In addition, TAR clones from BES1 (TAR40), BES2 (TAR129) and BES17 (TAR59) were compared to the same BES previously sequenced from a BAC library [27] and were 99%,100% and 99% identical, respectively, indicating that TAR cloning produced stable clones.

### BES architecture

Eighteen of the TAR clones contain full-length BESs, ending in telomeric hexamer repeats, and the 14 BES types show a striking conservation in overall structure and order of *ESAG*s (Figure 3). The 5′ end of each TAR clone contains the BES promoter that was used as the recombination target during cloning. Half of the BESs have a second promoter approximately 13 kb upstream from the first [28] which accounts for the differences in clone sizes observed in some of the BES groups. The region between the two promoters encodes *ESAG10* and a variety of pseudogenes that are commonly found in *T. brucei* subtelomeric regions (Figure 3). The presence of two promoters in some BES was confirmed by restriction mapping of the *NEO*-tagged clones. BES5 and BES8 were the only two discrepancies: *NEO*-tagged clones suggested the presence of 2 promoters, whereas TAR clones only had one, presumably because the downstream promoter was the target of the TAR cloning (Figure S3B). Downstream of the second promoter, the majority of sites encode polymorphic variants of *ESAG7*, *ESAG6*, *ESAG5*, *ESAG3*, *ESAG4*, *ESAG8*, *ESAG3*, *ESAG2*, *ESAG11* and *ESAG1*, in this conserved order. The only exception was an inversion between *ESAG7* and *ESAG6* in two BES groups (TAR29/BES12 and TAR56/BES13).

Nine BESs encode at least one copy of each *ESAG* (excluding *ESAG10*). We also observed frequent duplication of *ESAG*s, including a large-scale duplication in BES1 (TAR40), an ESAG7 triplication in BES4/TAR28 and three cases of divergent additional copies of *ESAG8* (BES1, BES13 and BES15).

The terminal *VSG*s are flanked upstream by tracts of 70-bp repeats, whose lengths vary in our assemblies between 0.2–7.1 kb, although this repeat region was only manually finished in 10 clones (Figure 3). The distance between the *VSG* and the telomeric repeats could be assessed in 17 TAR clones and ranged from 200–1590 nucleotides.

As described above, the analysis of the *NEO*-tagged clones was consistent with the analysis of TAR clones except in one case. Although VSG221/427-2 is a single-copy gene (Figure 1), we found it in both BES1/TAR40 and BES16/TAR128. The presence of VSG221/427-2 in BES1 has been firmly established from sequencing one BAC and two TAR clones. The promoter-proximal region (approx 40 kb) of TAR128 is identical to TAR126. Taken this data together, we conclude that TAR128 may be an artefact that resulted from recombination between BES1 and BES15. PCR analysis of additional TAR clones of BES16 failed to detect VSG221/427-2 (data not shown), further suggesting that TAR128 is not representative of BES16.

### Non-functional and novel BES genes

A defining feature shared by most BESs is the strict conservation of the 5′ copy of *ESAG3* and *ESAG11* as pseudogenes (Figure 3). The predominance of the latter is corroborated by a previous study describing the characterisation of this gene family [7]. In addition to these two pseudogenes, single frameshifts cause the presumed inactivation of *ESAG4* and *ESAG5* in some BESs. An analysis of *ESAG* processing signals gave further indication that some of the *ESAGs* may not localise correctly within the cell, including a number of *ESAG4* genes which lack sequences for surface targeting.

In five BESs (BESs 1, 2, 4, 5, 7) we identified a novel highly conserved putative protein coding sequence with a predicted signal peptide that we have designated ESAG12 (Figure 3). No functional clues could be discerned from sequence similarity or protein domain search results. A single homologue is found in the TREU 927 genome (Tb927.7.7510), although this is likely to be an under-representation due to the lack of subtelomeric sequence in the current TREU 927 genome assembly [18].

### Some BES deviate from the overall conserved structure

Several BESs diverge from this conserved structure either by encoding additional sequences or because they represent truncated sites, lacking members of individual *ESAG* families. Most BES harbour only the *VSG* between the 70 bp-repeat array and the telomere (Figure 3). BESs 2, 4, 7 and 15 are exceptions to this rule, showing additional sequences (*ingi* element, *VSG* or *ESAG3* pseudogene). A further three sites encode duplicated regions of 70-bp repeats (BES1, 14 and 15), in each instance sandwiching *VSG* pseudogenes (see below). Five of the BES groups do not contain one or more of the expected *ESAG* repertoire. Amongst this set are:

BES7 (TARs 65,153) lacks *ESAG7*, but contains tandem copies of *ESAG6*, with different hypervariable regions, with *ESAG6a* resembling an *ESAG6*/*ESAG7* mosaic. This BES could be activated *in vitro* at the same frequency as other BESs. In order to investigate whether any DNA rearrangements have donated a copy of *ESAG7* to the newly activated BES7, DNA from cells with an active or silent BES7 were compared by PCR and restriction mapping (Figure S2). No differences were observed, suggesting that a BES lacking *ESAG7* can indeed be activated, confirming previous observations [29].

BES8 (TAR64) is unusually small and only contains a single copy of *ESAG7* followed by the 70-bp repeat region and the *VSG* (Figure 3). Long-range analysis of chromosomal DNA from a BES8-tagged clone digested with *Apa* I supported this finding, with the *NEO and VSG427-14*-hybridizing restriction fragment being only ∼20 kb in length (Figure S3A). Genotyping of the NEO-tagged clone confirmed the presence of ESAG7 and absence of *ESAG6* (data not shown). Attempts to activate this BES in two independent clones were unsuccessful, suggesting that this mini-BES may not be functional.

BES10 (TAR134) lacks the central portion (*ESAG* 4-8-3-2-11-1). TAR134 also encodes only a 23-bp fraction of a 70-bp repeat. This site was not among those that were tagged with a drug resistance marker, so its potential for activation could not be assessed.

BES14 (TAR10), also lacks the *ESAG4*-*8-3* gene cluster and has two *VSG* pseudogenes sandwiched between 70-bp repeat arrays. An inconsistency was found between the NEO-tagged clone and TAR sequence. The *NEO*-tagged clone did not harbour the *VSG* predicted by the TAR clone. Instead, the *NEO* tagged cell line expressed *VSG427-8* upon activation, rather than *VSG427-19*, as predicted by the TAR sequence. We confirmed by PFGE that the BES activation did not involve gene rearrangements (data not shown), indicating that *VSG427-8* was at the BES14 locus prior to activation. Further examination of the TAR clones in the BES14 set revealed TAR117 to correspond to the *NEO*-tagged clone. An interesting consequence of these results is that two BESs (BES12 and BES14) contain the *VSG427-8* gene (Figures 1 and S1A).

In BES11 (TAR122), the *ESAG2-11-1* cluster has been replaced by a *VSG*-related gene (VR, Figure 3) and a full-length copy of *ESAG9*, the only copy found to date in a *T. brucei* BES. The *VR* gene is believed to be a *VSG* that has already evolved a new function and, like other *VR*s, has no 70-bp repeat sequences in its upstream flank [30]. Similarly to BES10, this site was not tagged with a drug selectable marker and therefore, activation *in vitro* could not be assessed.

BES12 (TAR29) has a deletion of the *ESAG4-8-3* cluster, but successful tagging and activation demonstrated that this site was functional *in vitro* (Table 1).

### Investigating the forces driving BES diversity

In order to investigate the role of recombination in the evolution of bloodstream expressions sites, we examined changes in phylogenetic relationships along the BESs using two methods.

#### Likelihood comparisons of optimal and constrained trees

**Figure 4:**
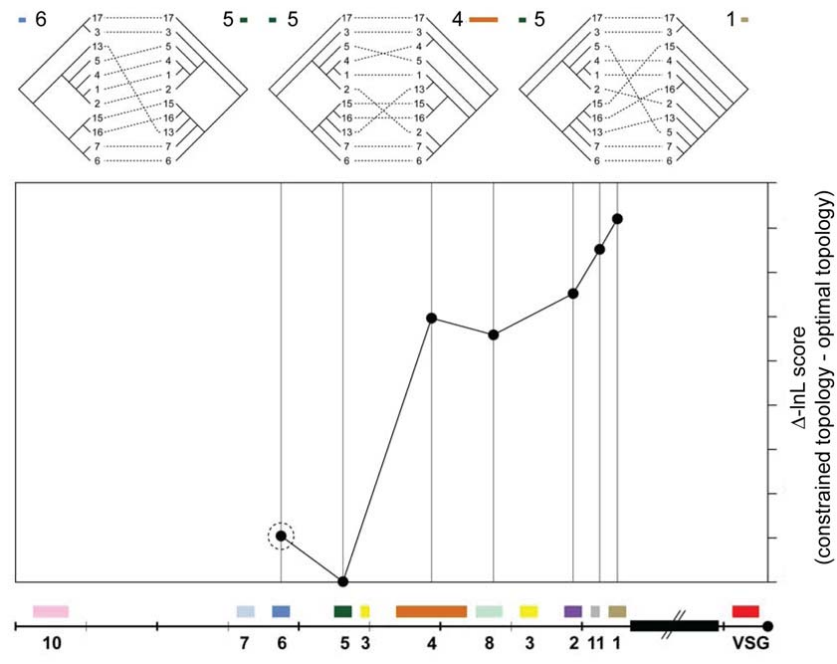
Quantifying the difference in phylogenetic signal along the bloodstream expression site. A. Three tanglegrams relate the *ESAG5* phylogeny with those of *ESAG6* (close match), *ESAG4* (moderate incongruence) and *ESAG1* (severe incongruence). Dashed lines link corresponding expression sites in each tree. Incongruence between trees increases from left to right. B. Comparison of likelihood scores between optimal and constrained tree topologies for *ESAG5*. The phylogenies of six other *ESAG* loci (indicated by a cartoon of the expression site) were used to constrain the estimation of the *ESAG5* tree; the difference in likelihood score between each of these constrained trees and the optimal *ESAG5* tree is plotted along the expression site. Non-significant differences in likelihood are denoted by a dashed circle as evident when constraining the *ESAG5* topology with the *ESAG6* topology; however, enforcing the topologies of central loci (*ESAGs 4* and *8*) caused a moderate decrease in likelihood, whilst constraining with *ESAG*s *2*, *11* or *1* caused a larger decrease.

There were obvious discrepancies between the optimal phylogenies for different *ESAG* loci, suggesting that the phylogenetic signal varied along the BESs. This was manifest in both the different shapes of ESAG trees and comparisons of their log-likelihood scores (Figures 4 and S4). Comparing the likelihood score of an optimal topology for any given *ESAG* with the score obtained for a tree constrained with the signal of another *ESAG* locus, typically indicated a decrease in likelihood and therefore a decreased probability of shared histories. Indeed, the drop in likelihood was generally proportional with physical distance, such that topologies from different ends of the expression sites were most dissimilar (Figures 4 and S4). Although topological differences between the phylogenies of neighbouring *ESAG*s were observed, these were non-significant and fell within the margins of systematic error. In fact, comparisons for all genes (Figure S4) showed that *ESAG*s fell into three clusters with regard to phylogenetic signal: *ESAG*s 7, 6 and *5*, *ESAG*s *4* and *8*, and *ESAG*s *2*, *1* and *11* with compatible phylogenetic signals within, but not between, these clusters.

#### Recombination analyses

To examine the fine scale structure of recombination within the BESs, a genetic algorithm-based tool (GARD) [31] was used to infer the numbers and locations of recombination breakpoints. Although this method relies on phylogenetic incompatibility to deduce recombination, GARD differs from the constrained tree analyses described above in two important respects: (i) the locations of putative breakpoints are inferred rather than fixed *a priori*, allowing us to characterize recombination within individual loci, and (ii) GARD is able to detect gene conversion events that affect branch lengths but not tree topology. When applied to the nucleotide alignments of each *ESAG* locus and to five intergenic regions, this method identified a large number of recombination tracts with distinct phylogenetic histories (Tables S1 and S2). Mean recombination tract lengths ranged from 90 bp/tract for *ESAG1* to 687 bp/tract for *ESAG10*, and mean tract lengths were similar in both coding and non-coding sequences. The GARD analyses demonstrate that phylogenetic breakpoints occur throughout the expression sites, consistent with frequent genetic exchange.

Phylogenetic inference of recombination can be misled by substitution rate variation, as could occur if different genes or regions within a gene are subject to different selection pressures. Therefore, a summary statistic-based approach, the Pairwise Homoplasy Index (Φ) Test [32], which has been shown to be robust to substitution rate variation, was applied to each alignment to validate the GARD analyses, and to each of the individual recombination tracts inferred by GARD to identify any further recombination that might exist. With the exception of *ESAG10*, which is highly conserved, significant values of Φ were obtained for each *ESAG* alignment, consistent with recombination within these loci (Table S2). In addition, the Φ values were significant in at least some of the GARD-inferred tracts within all *ESAG* loci except *ESAG8* and *ESAG10*, and in 30% of individual tracts overall. These results indicate that there are additional breakpoints not detected by GARD, and support the conclusion that there is extensive recombination throughout the entire BES.

#### Evidence of molecular adaptation within ESAG families

The role of selection in the evolution of bloodstream expression sites was examined by estimating the ratio of the non-synonymous and synonymous substitution rates (ω) within each *ESAG* family. A likelihood ratio test was used to assess whether the distribution of ω across codons is best explained by a model (M1a) which assumes that all sites are evolving neutrally or under purifying selection, or by a model (M2a) which includes an additional category of sites under positive selection (i.e., ω>1). As indicated by the higher likelihood scores of model M2a (Table S3), these tests provide significant evidence of adaptive evolution within *ESAGs* 4, 5, 6, and 7, and are nearly significant (p<0.1) for *ESAG1* and *ESAG3b*. In contrast, there is no evidence of adaptive evolution in *ESAGs* 2, 8 and 10. Selective pressures at individual codons were inferred using a Bayesian approach with model M2a used as a prior distribution on ω. As expected, the majority of sites appear to be subject to strong purifying selection (ω≪0.5), while the numbers of positively-selected sites corroborate the gene-wide analyses, with *ESAGs* 4–7 displaying widespread adaptation (Table S4). Taken together, these results indicate that all *ESAGs* are under strong purifying selection, presumably to maintain functionality, and that most *ESAGs* contain at least some sites which have undergone adaptive evolution over the course of BES differentiation.

## Discussion

The elucidation of the BES repertoire of a single *T. brucei* strain has produced a global view of BES structure and evolution. We have sequenced 19 out of 182 clones of BESs from *T. brucei* 427, chosen to represent as many different BESs as possible based on the preliminary analysis of the original set [19] and including duplicates. Sequence analysis identified 14 distinct BESs, one of which exists in two copies, giving a total number of 15 BESs. Have we found all of the BESs in *T. brucei* 427? Assuming that the promoter sequence is conserved in all BESs, we estimated the chance of missing any BESs in the entire library by simulating samples of 182 randomly sampled clones from populations with different numbers of underlying BESs. In such a simulation, no sites are ‘missed’ if there were 19 or fewer BESs. Based on all of the available data the subset chosen for sequencing appears to cover most, if not all, BESs. Thirteen of these 15 BES were tagged with the Neomycin resistance gene in 30 random integration events. Assuming equal probability of tagging any end we expect to miss at least 2 out of 15 BESs in 30 tagging events 65% of the time (simulation of 1000 random samples of 30 tagging events). No BESs were tagged that were absent from the collection of TAR clones and we therefore are confident that the library contains the entire *T. brucei* Lister 427 BES repertoire. We will however only be able to address the question as to whether the size of the BES repertoire of *T. brucei* Lister 427 is shared by other *Trypanosoma brucei* isolates once a large number of strains are analyzed. The genome strain *T. brucei* TREU 927 has fewer BESs based on hybridization analysis [33] and a similar set of *T. brucei* 927 BES TAR clones with only 5–8 promoter and *ESAG6* region sequence types have been identified (Becker and Louis, unpublished). In addition to having fewer BESs, *T. brucei* 927 also has smaller *VSG* basic copy arrays compared with *T. brucei* 427, though the genome strain *T. brucei* TREU 927 has one of the smallest genomes analysed among *T. brucei* isolates [34]. This makes it likely that the relatively large number of BESs that we observe here with *T. brucei* 427 does indeed fall within the range of BES copy numbers that may be observed among different isolates.

The validity of the TAR cloning is demonstrated by the identical sequences obtained from two independent TAR clones of the same BES, by the comparison to previously published sequences and by the congruence with the data obtained from the NEO-tagged BES. Given both the number of TAR clones from known BESs and TAR cloned DNA that does not exist in the genome, we get a maximum estimate of 15% of aberrant TAR clones in the original library. In fact, only one of the 19 TAR clones selected for sequencing (TAR128) is inconsistent with other data and could be a recombinant between two BESs generated during TAR cloning. The other clones originally classified with this clone in BES group 16 appear not to be recombinant and could be sequenced in the future.

The sequencing effort has also highlighted the need to return to the original TAR clone set to account for misclassifications based on the hypervariable region of *ESAG6* and the promoter sequence. Given that the promoter sequence was the target for recombination, the actual cloning procedure may have introduced variability in this region not present in the genome. As a consequence, two TAR clones were re-assigned in this study to different BES groups, giving fewer groupings than the original classification into 19 groups. It is clear that correct and complete characterisation of the telomeres requires a combination of saturation cloning and sequencing of well-chosen clones.

Characterisation of parasites with activated *NEO*-tagged BESs revealed that, as expected, *in situ* switching was the most frequent event. Interestingly, in three out of 28 switchers, we observed a switching mechanism, in which the previously active BES (BES1) was replaced by a duplication of the newly activated BES (7, 13 or 15b). Most likely recombination between the two subtelomeric regions occurs upstream of the drug-resistance genes, possibly at the promoter region, the 50 bp-repeat array, or in an even more internal chromosomal location. It is unclear which of the two BES copies is the active one. Nevertheless, our observations confirm what has been previously reported that an entire BES can be duplicated and transposed to a different locus [23], [24], which is reminiscent of break-induced replication in yeast. In fact, when a double-strand break is induced in subtelomeric regions of *S. cerevisiae*, that end is often replaced by a duplicated copy of another end (reviewed in [35]). Such mechanism could explain the existence of two copies of BES15 and it may also have contributed to the large number of BES in *T. brucei* 427 relative to other isolates.

The BESs presented here show considerable diversity in the *ESAG* complement, though there is an overall conservation of order that agrees with the generic BES structure previously proposed [27] (top diagram in Figure 3). There are some clear exceptions within the overall conservation, ranging from a small BES containing only *ESAG7* and a *VSG*, to a BES containing *ESAG* duplications and triplications. The BES-tagging data presented here redefine the requirement for a minimal BES, which appears only to entail functional copies of *ESAG*s 1, 2 and 6, although *ESAG1* is not essential within an active BES [16], [17] and *ESAG2* is absent from the BES encoding *SRA*, the *T. b. rhodesiense* gene able to confer resistance to human serum in infections [36]. There appears to be no requirement for *ESAG7*, as previously postulated [27]. The smallest BES we came across as part of this study only encodes *ESAG7* in addition to *VSG* (BES8). We were unfortunately unable to show activation of this site *in vitro* but it may yet indicate that none of the *ESAGs* need to be expressed from the active BES. However, the absolute requirement of *ESAG*s in an expression site is difficult to test because low-level transcription of *ESAG6* and *ESAG7* and indeed other *ESAG*s may occur from ‘silent’ sites [37], [38]. Even if these truncated BES are not functional, they may still contribute sequences via gene conversion to other BESs.

One surprising finding was the presence of additional sequences downstream of the 70-bp repeat in some BESs, as well as the number of BESs containing additional *VSG* pseudogenes. The presence of pseudogenes towards the downstream end of the BES is not entirely unexpected though, as this area is a recombinational hotspot, continually accepting incoming copies of *VSG* cassettes in order to mediate antigenic variation. As the recombination processes involved may not be constrained by the high stringency normally imposed by mismatch repair [39], it has to be expected that there will be errors, such as a duplicated cassette being copied into another cassette, rather than replacing it. Most of the *VSG*s in the silent archive are full-length pseudogenes, at least some of which contribute ‘healthy’ sequence to novel mosaic expressed *VSG*s [30]. It has been speculated that silent BESs might safely foster the stepwise assembly of mosaics, in contrast to the active BES, from which there is an absolute requirement for expression of an intact *VSG* [40], [41]. The present findings are compatible with this proposal, but not conclusive. The *VSG* pseudogenes could be copies of silent *VSG* pseudogenes, or partially assembled mosaics, or genes that have degenerated *in situ* through lack of requirement for expression. Work is underway to address the question of mosaic *VSG* assembly and expression.

The repertoire of BESs gives some indication of their proliferation among telomeres and their macroevolution. The driving forces that have shaped this repertoire are segmental duplications and deletions, recombination events typical of subtelomeres. However, gene content does not always coincide with the relationships among ESAG genes; for instance, BES13, 15 and 16 all possess a tandem duplication of *ESAG8*, but are not closely related based on their *ESAG8* nucleotide sequences. The incongruence between gene trees for the different *ESAG*s confirms that BESs have not diversified through ‘orthodox’ patterns of duplication and divergence. Rather, each BES is a mosaic and the product of frequent recombination between telomeres; this may occur through reciprocal crossover events between homologous strands at mitosis, or through non-reciprocal gene conversion. Recombination is frequently non-homologous, as we observe both segmental deletions and duplications, (for instance the substantial duplications and triplications within BES1), and variations in length, with some BESs greatly reduced in size (e.g. BES8 and BES10).

This process is consistent with previous observations of ‘telomere exchange’ [42], though the prevalence of mosaics demonstrates that whole telomeres are not typically exchanged. Instead, the data suggest that clusters of contiguous *ESAG*s share common histories, and therefore have not frequently been split by recombination. There appears to be a physical limit on the frequency of recombination breakpoints, which seldom occur in between *ESAG*s *7*, *6* and *5*, *ESAG*s *4* and *8* or between *ESAG*s*2*, *1* and *11* (Figure 4, Table S2). However, the transposition of an isolated ESAG7 copy into an active expression site (without ESAG6 and 5) has been reported under *in vitro* drug and serum selection pressure [38]. This provides experimental evidence that *ESAG*s can and do move between sites, with the loss of a particular allele from the active BES; our data qualify this by showing that *ESAG7* usually transpose in unison with *ESAG*s*6* and *5* (otherwise they would not share a common phylogenetic history).

Given that frequent breakpoints coincide with the position of *ESAG3* loci between linkage blocks, these could play a role in orientating expression sites prior to the exchange of intervening regions. This could also explain why *ESAG3a*, *b* and *c* sequences do not cluster by position (data not shown). It is therefore conceivable that the conserved, repetitive, higher order structure of expression sites may be maintained as a template for recombination, either to enhance the function of *ESAG*s, or of the VSG switching mechanism. Alternatively, the overall structure could be a secondary, non-adaptive result of the *VSG* switching events.

The majority of *ESAG*s within expression sites are unusual in that they are part of larger gene families present at chromosome internal locations within the genome but ‘behave’ like a tandem gene array or highly linked loci [43]. Internal and telomeric gene copies seem to evolve in very different ways. Internal copies of some families, for example *ESAG4* and *5* are more diverse and retain orthology with corresponding loci in the related species. Telomeric *ESAG* copies in contrast have relatively low levels of variation and therefore always form a clade in *ESAG* family phylogenies (data not shown). This indicates that frequent recombination between telomeric copies leads to homogenisation and concerted evolution of *ESAG* families and both GARD and PHI analyses have shown that most *ESAG* alignments contain chimaeras (Figures 4 and S5, Table S1). In addition, localised diversification has clearly occurred in some *ESAG* families such as *ESAG7* and *6*, where the most diversifying codons can be mapped to the exposed surface of the molecule, including the hypervariable region. [11] (M. Carrington, University of Cambridge, personal communication).

We also postulate that a by-product of the recombination between *ESAG* families is the prevalence of some *ESAGs* as apparent pseudogenes. Like *VSG*s, *ESAG* copies may be routinely corrupted but continue to provide genetic resources for functional copies, since they can be rescued by subsequent gene conversion events. Whether *ESAG* evolution is adaptive, or simply a secondary consequence of sharing the expression site environment, remains to be seen.

The conclusion that both the whole BES and individual *ESAGs* are mosaics raises the question as to how it is possible for two contiguous markers, such as *ESAG1* and *2*, to display parallel evolutionary histories, when both gene families are being homogenised by frequent gene conversions. This should effectively randomise the relationships of each ESAG family relative to each other. Essentially, we could be observing recombination on two different scales. Entire BESs recombine, perhaps as a secondary effect of the *VSG* switching mechanism, but this is limited by spatial proximity. At a finer scale, individual *ESAG*s recombine with paralogs in other expression sites, which restrains the normal process of divergence, tending to homogenise the entire family. Our observation that BESs retain phylogenetic signals, i.e. a pattern of divergence, demonstrates that substitutions affecting *ESAG* sequences are not entirely homogenised by recombination. Therefore, for phylogenetic signals to persist, the substitution rate, perhaps augmented by positive selection, must limit the extent of homogenisation through recombination in a dynamic equilibrium. Neighbouring *ESAG*s that are not separated by recombination share this equilibrium and therefore, phylogenetic signal.

This work opens important perspectives in understanding the molecular mechanisms of antigenic variation and telomere biology. The fact that the short BES8 and BES10 could not be activated *in vitro* opens the possibility of using these sites to identify the minimum requirements to make a functional BES. The role of telomeres in BES transcription has been extensively studied by several groups (reviewed in [44]). Truncated BES may be a useful tool to study telomere biology in *T. brucei*, since the distance between the promoter and the telomere is much shorter. Finally, BES sequences and overall architecture will aid the identification of the mediators involved in *VSG* switching. Such studies may reveal mechanisms that are relevant to telomere biology and antigenic variation in other important pathogens.

## Materials and Methods

### *Trypanosoma brucei*

The BES TAR library was constructed from *T. brucei* Lister 427 strain clone 221a [45], in which a hygromycin-resistance gene had been previously introduced immediately downstream of the promoter of the active VSG221/427-2 expression site and a neomycin-resistance gene downstream of the promoter of the silent VSGVO2 expression site [22]. BES-tagged cell lines were also derived from wild-type *T. brucei* 221a. Bloodstream-form trypanosomes were grown in HMI-9 medium [46]. Stable transfections were performed using the BTX Electroporator [47].

### Isolation of TAR clones and preparation of sequencing libraries

Yeast artificial chromosomes (YACs) containing BESs, TAR-cloned using the conserved *VSG* BES promoter as a recombination targets, have been previously described [19]. A representative clone was chosen from each group, selecting a larger sized clone wherever possible. Clones were isolated from yeast transformants through separation of the YAC DNA from endogenous chromosomes by CHEF (contour clamped homogeneous electric field) gel electrophoresis [48]. The recovered DNA was fragmented by sonication and end-repaired with mung bean nuclease prior to preparative agarose gel electrophoresis. DNA fragments of 2–4 kb and 1.4–2 kb were purified and cloned into pUC18 plasmid vector.

### Sequence determination, assembly and annotation

Nineteen TAR clones were sequenced by random sequencing of small insert libraries using dye-terminator chemistry on an ABI 3730 sequencing machine. Sequence reads were assembled using Phrap [www.phrap.org; P. Green, unpublished)]. Manual base calling and finishing was carried out using Gap4 software (http://www.mrc-lmb.cam.ac.uk/pubseq/manual/gap4_unix_1.html). Gaps and low quality regions of the sequence were resolved by primer walking and targeted polymerase chain reactions (PCR). A number of gaps, all exclusively located in the 70-bp repeat regions, remain in the assemblies and are indicated by 100 N's (Figure 3).

Each clone was annotated using the Artemis software [49]. Protein coding sequences were predicted as previously described and analysed to assign putative functions as previously described [18]. The full annotation of 19 expression sites can be viewed and searched via GeneDB (http://www.genedb.org/genedb/tbrucei427/) and sequences have been submitted to EMBL with the following accession numbers: FM162566 - FM162583.

Current VSG nomenclature reflects multiple naming schemes. We therefore propose a new systematic nomenclature based on sequential numbering with reference to the *T. brucei* Lister 427 strain (Table 1).

### Sequence alignments

The Needle program, an implementation of Needleman-Wunsch algorithm in the EMBOSS [50] software package was used to perform global alignments using default parameters. The region of the imperfect 70-bp repeats was excluded from each alignment. Local alignments were performed using ClustalW (http://www.ebi.ac.uk/clustalw/index.html) with default parameters

### Generation of *T. brucei* clones with tagged BES

BES-tagged clones were generated by introducing a Puromycin resistance gene (*PUR*) downstream of the promoter of a wild-type *VSG221/427-2* expressing cell line. *NEO* was subsequently integrated downstream of the promoters of random BESs conferring resistance to G418. Clones obtained from this transfection were named BF-LF17.x or BF-LF18.x. depending on the presence or absence of the 50-bp repeat array upstream of BES1 promoter (Figueiredo *et al.*, unpublished) (see also Data S1).

### BES switching assay

To prevent premature growth of *in situ* switchers, *NEO*-tagged clones were grown under Puromycin selection. After 5 days, cells were subcloned by limiting dilution in the absence of any drug to allow independent switching in each subclone. 2.4×10^7^ cells of 2–5 subclones were subsequently diluted in HMI-9 containing 100 µg/mL G418, and distributed in a 24-well plate. After 6–8 days, the number of G418-resistant clones was counted. Switching frequency was calculated by dividing the number of G418-resistant clones by the total number of plated cells. One or two clones from each plate were tested for Puromycin sensitivity by diluting 10^5^ cells in 5 mL of HMI-9 containing Puromycin at 1 µg/mL. Cell growth was scored after 2 days. Western Blotting was performed to confirm that G418-resistant cells no longer expressed VSG221/427-2.

### Cloning the *ESAG6* hypervariable region

The *ESAG6* hypervariable region of each *NEO*-tagged BES was PCR amplified using a *NEO*-specific primer and one of two *ESAG6*-specific primers (5′-TAAAGAGAGTTGTTCACTCAC-3′ or 5′-TGTTCACTCACTCTCTTTGAC-3′) and the products gel-purified and sequenced. From clones with two consecutive *ESAG6* genes, the *ESAG6* hypervariable region from the most telomeric *ESAG6* gene, was reamplified from the largest PCR fragment by nested-PCR with primers: 5′-CACTAATGATCAGCTTTACG-3′ and 5′-GACTCTTTTACACGTGAATC-3′. The resulting 1-kb fragment was purified and sequenced.

### Cloning the expressed VSG

Total RNA was extracted from ∼10^8^ G418-resistant cells with RNA STAT-60 reagent (TEL-TEST, Inc.), following the manufacturer's instructions. cDNA was synthesized using a PolyT-primer and the StrataScript™ First Strand Synthesis System (Stratagene). *VSG* cDNA was amplified with primers that bind to the spliced leader (5′-GACTAGTTTCTGTACTATAT-3′) and to a conserved sequence found in the 3′UTR of all *VSG* (5′-GTGTTAAAATATATC-3′). Using the spliced leader primer ensured that the amplified product originated only from *trans*-spliced RNA and not from a DNA contaminant. PCR products were subsequently cloned in pGEM-T Easy (Promega) and sequenced.

### Chromosome separation by CHEF

*T. brucei* chromosomes were separated using a CHEF electrophoresis apparatus (CHEF-DR III, Bio-Rad), loading DNA from approximately 2×10^7^ cells per lane. Electrophoresis conditions for the panel separating *T. brucei* intermediate chromosomes were: 25 s pulse time, 6 V/cm, for 20 h, in 0.5×TBE, at 14°C, using a gel of 1% high-strength agarose (Helena Biosciences). *T. brucei* megabase chromosomes were separated using 1400–700 s linear ramped pulse, 2.5 V/cm, for 144 h in 1×TBE at 14°C in a gel with 1.2% high-strength agarose.

### Long-range Southern blotting

Agarose-embedded chromosome plugs were digested with 40 units of *Apa* I or *Apa* I and *Xma* I (New England Biolabs), as previously described [51]. Restriction products were separated on a 1.0% agarose gel in 0.5×Tris-Borate-EDTA buffer (TBE) in the Stratagene Rotating Agarose Gel Electrophoresis variant of Pulsed Field Gel Electrophoresis (PFGE) at 12°C and with a rotation angle of 120°. The running program for *Apa* I restriction fragments consisted of a 5 to 15 sec linear ramp at a constant 155 V for 18 h. For *Apa* I and *Xma* I restriction fragments the program was 1 to 4 sec linear ramp at a constant 150 V for 18 h. After alkaline transfer and hybridization with a radioactive probe, the Hybond-N+ Nylon membranes (Amersham Biosciences) were typically washed at 65°C as follows: twice for 20 min with 2×Sodium Chloride/Sodium Citrate (SSC), 0.1% SDS and twice for 20 min with 0.5×SSC, 0.1%SDS.

### Comparison of phylogenetic signal among *ESAG*s

ClustalX was used to align homologs of 7 ESAG loci from 11 different expression sites [52]. The maximum likelihood phylogenetic tree topology was estimated for each locus using the program PHYML [53], with a GTR+Γ model and parameters estimated from the data. By constraining the resulting trees with the optimal topology of each other locus in turn a further six tree topologies were produced for each locus. The differences in likelihood value between the optimal tree and each constrained tree were calculated and the significance evaluated using the Shimodaira-Hasegawa test [54] thus quantifying the difference in phylogenetic signal between different loci along the expression site. Such likelihood comparisons require that two trees have equal taxon sets. For this reason a consistent gene complement from each expression site was used and *ESAG10* and *ESAG7* were therefore excluded. *ESAG3* was also not used because it is present in three positions along the expression site and does not cluster by position; hence, its phylogenetic signal was not amenable to positional comparisons.

### Characterization of recombination

Sequence alignments of each *ESAG* family and selected intergenic regions were generated with ClustalX as described above, manually edited and gap-stripped. Each alignment was tested for evidence of recombination using two approaches. (i) The likelihood-based method implemented in the program GARD ([31]; http://www.datamonkey.org/GARD) was used to identify putative recombination and gene conversion breakpoints, employing a nucleotide substitution model specified by the HyPhy software package ([55]; http://www.hyphy.org). (ii) The program Phi [32]; http://www.mcb.mcgill.ca/trevor) was used to assess the significance of the pairwise homoplasy index (Φ_w_) with a window size of 100 bases and 10,000 permutations per test.

### Adaptive evolution of *ESAG* sequences

*ESAG* protein-coding sequences were analysed for adaptive evolution by estimating the relative rates of non-synonymous and synonymous substitutions within each ESAG family. Positive selection is indicated by a non-synonymous/synonymous rate ratio (ω) greater than 1. The method was modified for recombinant sequences by allowing the genealogy to vary across the alignment while sharing the parameters of the codon substitution model between tracts [56]. The dimensions and genealogies of putative recombination tracts were provided by the GARD analyses described previously. HyPhy was used to obtain maximum likelihood estimates for two nested models of the distribution of ω. A likelihood ratio test for positive selection was performed with a χ^2^-distribution on two degrees of freedom by comparing twice the log-likelihood difference between a nearly-neutral model (M1a, in which ω cannot exceed 1) and a selection model (M2a) that included an additional category for ω>1 [57]. Evidence for adaptive evolution at individual codons was tested using an empirical Bayes method [57]: model M2a, with the maximum likelihood estimated parameters, was taken as a prior distribution for ω, and Bayes' formula was used to calculate the posterior distribution of ω at each codon, given the sequence data.

## Supporting Information

Data S1 Additional Materials and Methods; Figure and Table legends of supporting data
(0.04 MB DOC)

Figure S1 PFGE Southern blotting.
(2.68 MB EPS)

Figure S2 Absence of ESAG7 from BES7.
(1.82 MB EPS)

Figure S3 Long-range restriction mapping of NEO-tagged clones.
(5.15 MB EPS)

Figure S4 Quantifying the difference in phylogenetic signal along the bloodstream expression site.
(1.97 MB EPS)

Figure S5 GARD analysis and interpretation using ESAG2 sequences as an example.
(5.49 MB EPS)

Table S1 Locations of breakpoints inferred by GARD analysis of ESAG alignments.
(0.38 MB EPS)

Table S2 Diversity and recombination within BES coding and intergenic sequences.
(0.45 MB EPS)

Table S3 Non-synonymous substitution rates for ESAGs.
(0.35 MB EPS)

Table S4 Positively selected codons in ESAG proteins.
(0.32 MB EPS)
