# Supplementary material for: Telomeric Expression Sites Are Highly Conserved in Trypanosoma brucei
Source: PLoS One. 2008 Oct 27;3(10):e3527. doi: 10.1371/journal.pone.0003527 (PMC2567434; doi:10.1371/journal.pone.0003527)
Supplement: Data S1 — Additional Materials and Methods; Figure and Table legends of supporting data (0.04 MB DOC) [file pone.0003527.s001.doc]

## **Supplementary Materials and Methods**

### Generation of tagged BESs

BES-tagged clones were generated by transfecting wild-type cells in two steps. The Puromycin resistance gene (*PUR*) was integrated 240 bp downstream of the BES1 promoter, using the pLF12 construct. pLF12 consists of three fragments cloned into pBluescriptSKII+ in the following 5´ to 3´ order: upstream promoter (UP), *PUR* flanked by the Aldolase splice acceptor site and 3´ untranslated region, and downstream promoter (DP). UP is the 1590-bp fragment between the 50-bp repeat array and 240 bp downstream from the ES transcription start site. UP was PCR amplified from a BES1-containing H25N7 BAC clone with the primers 5´-cgGGTACCtttatgttttgtgtattatttag-3´ and 5´-ccgCTCGAGatacgctcagcccgtc-3´, which introduced *Kpn* I and *Xho* I restriction sites respectively. DP is the 1150-bp region running from 287 bp downstream of the ES transcription start site to *ESAG7*. DP was PCR amplified from the same BAC clone with primers 5´-tccCCCGGgtcgatcgacgaagagc-3´ and 5´-ataagaatGCGGCCGCtttccgcttcgttactgag-3´, which inserted *Xma* I and *Not* I restriction sites respectively. The *PUR* cassette was obtained by digesting the pHD309-PUR plasmid [47] with *Xho* I and *Xma* I enzymes. pLF13 was obtained by digesting pLF12 with *Hin*d III and *Bam*H I and replacing *PUR* with *NEO*. The resulting cell line, BF-LF12.6, was subsequently transfected with pLF13, which integrated the Neomycin resistance gene (*NEO*) in an identical position of a second random BES. Clones obtained from this transfection were named BF-LF17.x or BF-LF18.x for clones with or without the 50-bp repeat array from BES1, respectively (L. Figueiredo et al, in preparation). Prior to transfection, 20 μg of pLF12 or pLF13 were digested with *Kpn* I and *Sac* I to release fragments of 3.7 kb or 3.9 kb, respectively. To select for integration of pLF12 into the actively transcribed BES1, we used high concentrations of Puromycin (up to 5 μg/mL). pLF13 integration into a random silent BES was achieved by selecting with low concentrations of G418 (2 μg/mL). To confirm that *NEO* was integrated into a BES, PCR amplification was performed with primers 5´-ATCGCCTTCTATCGCCTTC-3´ for *NEO* and 5´-gactcttttacacgtgaatc-3´, which matches a conserved sequence between *ESAG6* and *ESAG7*.

## **Supplementary Figures and Tables:**

### Figure S1

PFGE Southern blotting. A. *VSG427-*8 is present in BES12 and BES14. **B.** Two copies of BES15 harboring VSG427-11 are present on two different MBCs.

### Figure S2

Absence of *ESAG7* from BES7. *Xho* I and *Eco* RI restriction analysis of silent and active BES7 clones (17.9 and 17.9s2 respectively) is consistent with the absence of *ESAG7* from this BES. Restriction maps of other BES are shown for comparison.

### Figure S3

Long-range restriction mapping of NEO-tagged clones. A. BES8 is an abnormally small BES. Chromosomal DNA from several BES-tagged clones was digested with *Apa* I (A) and resolved by Long-Range RAGE. A *NEO* probe was used to visualize the specific restriction fragment from the tagged BES. BES4-tagged clone shows the smallest ApaI / NEO containing fragment (~23kb). This fragment co-hybridizes with VSG14. B. Restriction mapping of NEO-tagged BESs to test for the presence of ESAG10 and a second BES promoter. Chromosomal was digested with *Apa* I (A) and *Xma* I (X). *NEO* probe was used as above. As predicted from the TAR sequences (on the left), three classes of restriction fragments are detected: ~5, 15 and 50kb.

### Figure S4

Quantifying the difference in phylogenetic signal along the bloodstream expression site. For each of seven loci (*ESAGs* 1, 2, 4, 5, 6, 8 and 11), an optimal gene tree was estimated without any topological constraints. The tree was then rebuilt six times, each time applying the tree topology of a different *ESAG* data set as constraints on the search process. The difference in likelihood score between the optimal (unconstrained) and each constrained tree is plotted for each *ESAG* locus, starting from the 5´ end. Where the difference in likelihood scores was non-significant, (i.e., did not exceed systematic error), the data point is circled. All other data points represent incompatible phylogenetic topologies. For example, in the first panel the data set is provided by *ESAG* 6; when any other *ESAG* gene tree topology was used to constrain the estimation of the *ESAG* 6 tree, the likelihood of that tree was significantly lower (i.e., more negative) than the unconstrained tree. Only for *ESAG* 5 was this difference non-significant.

### Figure S5

GARD analysis and interpretation using *ESAG2* sequences as an example. A. Output of GARD analysis showing step-wise identification of breakpoints along the 1413-bp *ESAG2* sequence. Each breakpoint was inferred through comparison of the Aikake Information Criterion (D c-AIC) for phylogenetic models with and without one additional breakpoint. Distinct recombination tracts are differentially coloured. B. Sequence alignment of 11 BES for the region between 836 and 1137 bp, encompassing the breakpoints at 904 and 1042 bp in A. (marked by white arrows). C. Bayesian phylograms for the recombinant region (i.e., between breakpoints, centre), and for up- and downstream regions (left and right respectively). Numbers at nodes denote posterior probabilities. D. Consensus network estimated using SplitsTree4 of all tract trees in C.

### Table S1

Locations of breakpoints inferred by GARD analysis of *ESAG* alignments.

### Table S2

Diversity and recombination within BES coding and intergenic sequences.

CDS (coding) and IGS (intergenic) sequences; N = number of sequences in alignment; L = Length of BES 40 sequence (BES 15 for loci between the duplicated promoters); % SS = percentage of segregating sites; p = average pairwise diversity; # tracts = number of recombinant tracts inferred by GARD analysis; p-value of Pairwise Homoplasy Index test using 10,000 permutations.

### Table S3

Non-synonymous substitution rates for *ESAGs*.

Locations (w) and weights (p) of discrete priors for the relative rate of non-synonymous to synonymous substitutions under models M1a (2 atoms) and M2a (3 atoms); p-value of the difference in log-likelihoods between M2a and M1a under a c^2^-distribution with two degrees of freedom. Numbers of coding sequences used in each analysis are shown in parentheses beneath the gene family names.

### Table S4

Positively selected codons in ESAG proteins.

Locations of codons with posterior probabilities of (w > 2) greater than 0.5 (plain font) or 0.9 (bold font). All locations are given relative to the start of the reference sequence. Note that the prior distributions of w for ESAG8 and ESAG10 have no atom with w > 2.
